# Local adaptation to climate inferred from intraspecific variation in plant functional traits along a latitudinal gradient

**DOI:** 10.1093/conphys/coae018

**Published:** 2024-05-05

**Authors:** Emily P Tudor, Wolfgang Lewandrowski, Siegfried Krauss, Erik J Veneklaas

**Affiliations:** School of Biological Sciences, University of Western Australia, 35 Stirling Highway, Crawley, WA 6009, Australia; Department of Biodiversity Conservation and Attractions, Kings Park Science, 2 Kattidj Close, Kings Park, WA 6005, Australia; School of Biological Sciences, University of Western Australia, 35 Stirling Highway, Crawley, WA 6009, Australia; Department of Biodiversity Conservation and Attractions, Kings Park Science, 2 Kattidj Close, Kings Park, WA 6005, Australia; School of Biological Sciences, University of Western Australia, 35 Stirling Highway, Crawley, WA 6009, Australia; Department of Biodiversity Conservation and Attractions, Kings Park Science, 2 Kattidj Close, Kings Park, WA 6005, Australia; School of Biological Sciences, University of Western Australia, 35 Stirling Highway, Crawley, WA 6009, Australia

**Keywords:** Climate change, environment–trait relationships, intraspecific trait variation, morphology, physiology, soil, Stylidium

## Abstract

Ascertaining the traits important for acclimation and adaptation is a critical first step to predicting the fate of populations and species facing rapid environmental change. One of the primary challenges in trait-based ecology is understanding the patterns and processes underpinning functional trait variation in plants. Studying intraspecific variation of functional traits across latitudinal gradients offers an excellent *in situ* approach to assess associations with environmental factors, which naturally covary along these spatial scales such as the local climate and soil profiles. Therefore, we examined how climatic and edaphic conditions varied across a ~160-km latitudinal gradient to understand how these conditions were associated with the physiological performance and morphological expression within five spatially distinct populations spanning the latitudinal distribution of a model species (*Stylidium hispidum* Lindl.). Northern populations had patterns of trait means reflecting water conservation strategies that included reduced gas exchange, rosette size and floral investment compared to the southern populations. Redundancy analysis, together with variance partitioning, showed that climate factors accounted for a significantly greater portion of the weighted variance in plant trait data (22.1%; adjusted *R*^2^ = 0.192) than edaphic factors (9.3%; adjusted *R*^2^ = 0.08). Disentangling such independent and interactive abiotic drivers of functional trait variation will deliver key insights into the mechanisms underpinning local adaptation and population-level responses to current and future climates.

## Teaser

Results of this study show how the morphology and physiology of an endemic Australian plant respond to local climate and soil profiles across a ~160 km latitudinal gradient. Climatic factors accounted for more of the variation in functional traits than soil, suggesting that trait shifts might occur with climate change.

## Introduction

Anthropogenic activities are causing widespread and rapid changes to the earth’s climate, with weather extremes, such as frequent heatwaves, prolonged severe droughts and variability of precipitation events increasing (IPCC, 2021). These climate events are expected to have major consequences for the fitness and persistence of numerous species, with risks of severe spatial displacements and extinctions (Fletcher *et al.*, 2020; Miller *et al.*, 2020). Impacts associated with climate change are some of the most challenging threats to manage, given the global scale and the uncertainties of the responses of species and ecosystems to multifaceted drivers of environmental change (Millar *et al.*, 2007; Bellard *et al.*, 2012; Dube *et al.*, 2018). Additionally, there is growing need to understand how populations vary in their acclimation and adaptation to environmental change, and the risk of local populations not possessing the plasticity of phenotypic traits to effectively cope with changing conditions, or the genetic variability to adapt to them (Breed *et al.*, 2018; Harrison, 2021). Examining how populations vary in their responses to interacting environmental factors delivers key insights into the drivers of plant form and function, as well as the patterns and processes potentially underpinning species resilience or vulnerability to those environmental factors.

Trait-based approaches provide an empirical foundation for explaining patterns of variation and forecasting consequences of environmental change to ecosystems (Bjorkman *et al.*, 2018; Heilmeier, 2019). Functional traits can be considered as any morphological, physiological or phenological attribute of an organism, which can ultimately impact a measure of performance or fitness (Lavorel *et al.*, 2007; Violle *et al.*, 2007). These attributes can include indicators of plant form or function, such as plant size and photosynthetic rate (Cornelissen *et al.*, 2003; Perez-Harguindeguy *et al.*, 2016). Plant functional ecology aims to establish generalisable predictions across biological, temporal and spatial scales (Adler *et al.*, 2014), but calls have been made to better understand relationships between functional traits and the environment with explanatory and predictive power (Shipley *et al.*, 2016; Des Roches *et al.*, 2018). Established trait-based approaches have historically focussed largely on trait variation between species (McGill *et al.*, 2006). However, one of the primary challenges in trait-based ecology is accounting for and identifying the factors driving intraspecific variation in functional traits (Violle *et al.*, 2012). This is grounded in the knowledge that functional traits vary at the individual level and can have cascading effects across biological scales, which ultimately drives the functioning and persistence of populations and communities (Bolnick *et al.*, 2011).

Intraspecific trait variation (ITV) emerges from ontogenetic, phenotypic and genetic differentiation (Leimu and Fischer, 2008; Mason *et al.*, 2013; Henn *et al.*, 2018). The ability to produce different morphological and physiological responses to mitigate against environmental stress can increase the capacity for populations to persist into the future (Nicotra *et al.*, 2010; Araújo *et al.*, 2021). Greater ITV is likely to allow species to cope better with environmental stress through phenotypic plasticity in the short term, potentially promoting genotypic adaptation to future environmental norms in the long term (Nicotra *et al.*, 2010). However, research across several biological scales suggests that ITV is often constrained under environmental stress, such as warming, drying or nutrient limitation (Valladares *et al.*, 2007; Grassein *et al.*, 2010; Gugger *et al.*, 2015; Stotz *et al.*, 2021). Reduced intraspecific variation may also be a result of reduced genetic diversity, environmental filtering and strong stabilizing selection for certain traits, potentially leading to individuals being highly adapted to their local environment and improving their performance within a particular suite of conditions (Bradshaw, 1965; Schlichting, 1986; Schneider, 2022). Therefore, measuring the environmental factors that may drive trait variation can provide insight into how individuals are responding to current conditions, and how they may perform in the face of environmental change (Arnold *et al.*, 2019).

Selection pressures along environmental gradients often give rise to morphophysiological traits that show marked and predictable responses that are functionally linked to climatic and edaphic conditions along the gradient (Reich *et al.*, 2003; Dwyer and Laughlin, 2017; Kandlikar *et al.*, 2022). Climate factors, such as temperature and precipitation, impact the distribution and persistence of species through effects on cellular processes, reproduction and survival (Suzuki *et al.*, 2014). Similarly, edaphic factors, such as nutrient availability, acidity, salinity and metal toxicity, can also present critical limitations to plant physiology and growth, via impaired metabolic processes, enzyme activity and membrane stability (Ahanger *et al.*, 2016). Indeed, climatic and edaphic factors can also interact. For example, shifts in temperature and precipitation regimes can drive microbial activity and, by extension, decomposition rates, soil nutrient turnover, organic carbon (OC) stocks and nutrient availability (Lloyd and Taylor, 1994; Figueiredo *et al.*, 2018; Jansson and Hofmockel, 2020). More specifically, warming and drying can suppress microbial activity (Allison and Treseder, 2008), with nutrient limitations often linked to drier, lower rainfall systems (Moreno-Jiménez *et al.*, 2019). Despite their pervasive independent and interactive effects, there are few studies that simultaneously consider both climate and soil on intraspecific variation in plant functional traits *in situ* (Maire *et al.*, 2015; Simpson *et al.*, 2016; Joswig *et al.*, 2022). Nevertheless, understanding plant responses to local environmental conditions is crucial for guiding the conservation of extant populations, as well as the restoration of those that are degraded, which can maintain species functional diversity and ultimately biodiversity in the face of rapid environmental change.

**Figure 1:**
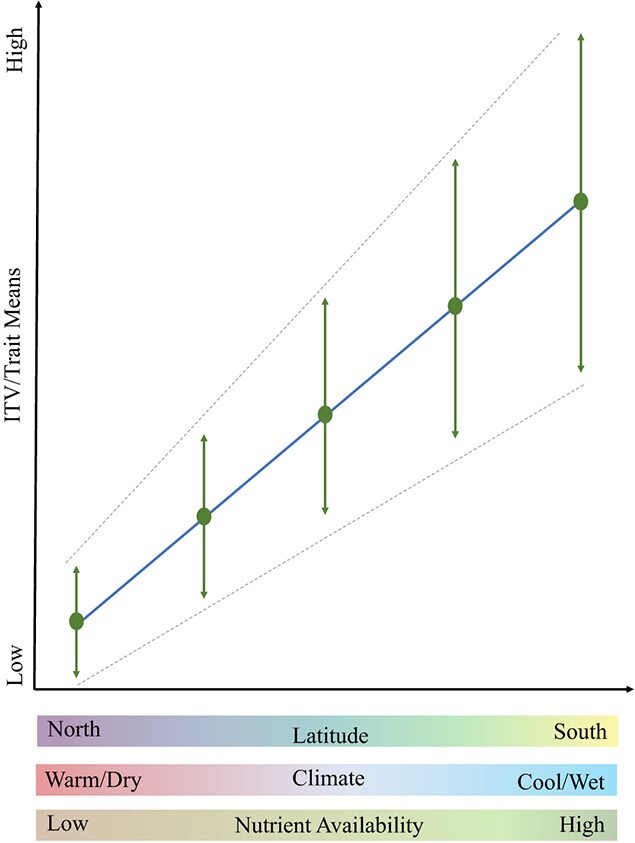
Conceptual diagram showcasing the hypotheses for change in intraspecific trait variation (ITV), calculated as the quartile coefficient of dispersion (QCD), and functional trait means for *S. hispidum* populations along the local latitudinal gradient. We expected greater ITV (represented by green arrows) in southern populations and for trait means to vary along the gradient (represented by green dots), with northern populations having trait means reflective of water conservation strategies (e.g. reduced stomatal conductance, smaller plant sizes and fewer flowers). Please note that not all functional traits were expected to exhibit the visualized positive trend across the latitudinal gradient (i.e. WUE was expected to decrease from north to south); the linear trend line is merely indicative of differences in means, irrespective of direction and slope.

Increased temperatures and declining rainfall in southwest Australia risk the ‘transition or collapse’ of the vulnerable Northern Jarrah Forest (NJF) ecosystems (Lawrence *et al.*, 2022). Therefore, this study assessed the physiological and morphological variation within and between five spatially distinct populations of the NJF endemic perennial, *Stylidium hispidum* (Lindl.), across a **~**160-km transect that spanned the latitudinal extent of the species distribution. We examined population differentiation in trait means and magnitude of trait variability along the latitudinal gradient and determined what proportion of morphophysiological variation was accounted for by the independent and combined effects of climate and soils. We set out to test whether northern populations would display reduced trait variability due to the hotter and drier conditions increasing the likelihood of abiotic stress (Fig. 1), as ITV is often constrained under environmental stress (Valladares *et al.*, 2007; Grassein *et al.*, 2010; Gugger *et al.*, 2015; Stotz *et al.*, 2021). Moreover, we expected trait means to vary along the gradient (Fig. 1), such that northern populations, exposed to warmer and drier conditions, would reflect water conservation strategies such as reduced stomatal conductance, greater water use efficiency (WUE), smaller plant sizes and fewer flowers (Descamps *et al.*, 2021; Valliere *et al.*, 2021), in contrast to southern populations. Given the southern populations are exposed to higher rainfall and cooler climate conditions, we expected that they experience higher nutrients availabilities and reflect traits associated with higher productivity (i.e. higher gas exchange rates, larger plant size, increased number of flowers).

## Materials and Methods

### Study species

*Stylidium* (Stylidiaceae), commonly known as trigger plants, is a large genus, comprising over 300 species distributed over a range of ecosystems and climate niches, mostly in Australia, with over 130 species occurring in the Jarrah Forest region of Western Australia (Western Australian Herbarium, 1988). The flowers of *Stylidium* are unique and feature a motile column composed of fused staminate and pistillate tissues that can be ‘triggered’ by touch. The swift movement is a pollination mechanism that deposits or retrieves pollen from insect pollinators such as bee flies (Diptera: Bombyliidae), hoverflies (Diptera: Syrphidae) and bees (Hymenoptera: Apoidea; Armbruster *et al.*, 1994).

*Stylidium hispidum* Lindl., or the White Butterfly Triggerplant, is a small perennial herb, ~12–28 cm tall, endemic to the NJF of southwest Western Australia and represents the focal species for this study. The species is a member of the *Stylidium piliferum* R.Br. complex and is characterized by a basal rosette with one or more floral racemes that emerge and flower during austral spring. Young plants are composed of a single rosette from an unbranched stem with green, linear, incurved leaves that may be tinged with pale maroon tones. Older plants typically feature up to eight tightly clustered rosettes, which are raised above the ground on stilt roots (Lowrie and Kenneally, 2017). The distribution of *S. hispidum* is restricted to a narrow transect parallel to the Darling Plateau with plants growing in highly weathered, lateritic soils. The genus has mycorrhizal associations (Brundrett and Abbott, 1991); however, it is unknown whether these mycorrhizas play a significant role in nutrient acquisition (Lambers *et al.*, 2018). Genetic analysis of spatially distinct populations of *S. hispidum* found significant differentiation among populations separated by at least 13–23 km along the latitudinal transect of distribution with 23% of the molecular variance partitioned among populations (Hufford *et al.*, 2016). In addition to the intrinsic conservation value of understanding how trait diversity is distributed across the distribution of a local endemic species, *S. hispidum* serves as a tractable representative from which meaningful conservation insights may be derived, especially for the many *Stylidium* that are already listed as ‘priority’ and ‘threatened’ species under the Biodiversity Conservation Act 2016 (Department of Biodiversity Conservation and Attractions, 2016).

**Figure 2:**
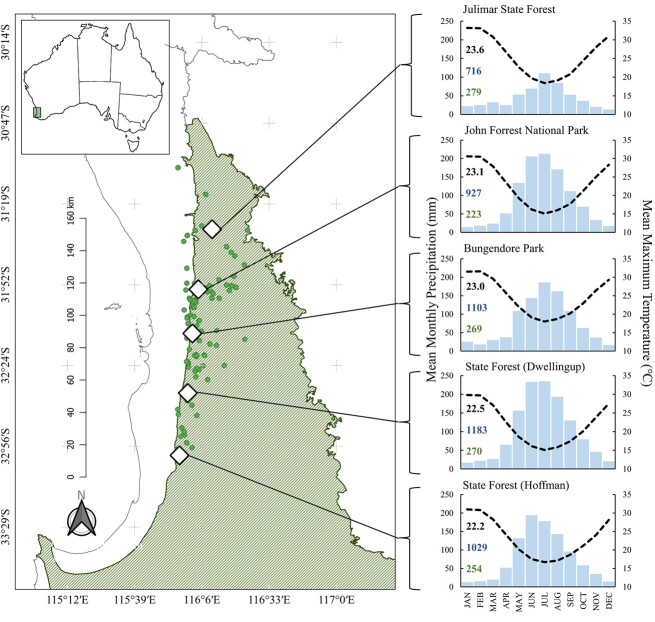
Location of study sites (◊) and climatologies distributed across the ~160-km latitudinal gradient along the Darling Scarp in the southwest corner of Western Australia (inset) with current distribution of *S. hispidum* populations (●) overlayed on the Interim Biogeographic Regionalisation for Australia with the NJF bioregion shaded in green. Monthly climate profiles composed of mean monthly precipitation (blue bars) and mean maximum monthly temperature (black dashed line) are constructed from the nearest available weather station data accessed from the Bureau of Meteorology, Perth, Western Australia. MAP (mm; blue text), mean temperature in the warmest quarter (°C; black text) and elevation (m; green text) are inset within each graph with precipitation and temperature data extracted from BioClim.

### Study area

Five study sites were selected along a **~**160-km north–south gradient of the NJF along the Darling Range spanning Julimar (−31.484691, 116.166501) in the north to Hoffman (−32.958298, 115.955832) in the south (Fig. 2). Sites were selected based on populations included in genetic analysis conducted by Hufford *et al.* (2016). The forest vegetation is composed of the dominant overstorey of *Eucalyptus marginata* Sm. and *Corymbia calophylla* (Lindl.) K.D.Hill & L.A.S.Johnson and a midstorey dominated by *Allocasuarina fraseriana* (Miq.) L.A.S.Johnson*, Banksia grandis* Willd., *Bossiaea aquifolium* Benth. and *Acacia* Mill. species, and a biodiverse understorey dominated by Asteraceae, Fabaceae, Restionaceae and Proteaceae species (Gardner and Bell, 2007; Koch and Samsa, 2007). Broadly, the region is characterized by a dry Mediterranean climate that presents hot dry summers and cool wet winters. Sites in the north experience a mean annual rainfall of ~700 mm compared to ~1200 mm in the south (Fig. 2), while the mean maximum temperatures are approximately 1.5°C higher in the northern sites compared to the southern sites (Fig. 2: Bureau of Meteorology, Perth, Western Australia). The NJF persists in an old, climatically buffered, infertile landscape (OCBIL; Hopper, 2009; Hopper *et al.*, 2021). The soils have developed on ~2.6 billion-year-old granite-gneiss metamorphic batholith (Nemchin and Pidgeon, 1997; Anand and Paine, 2002) and comprise well drained, shallow to moderately deep gravelly sands overlaying lateritic duricrust. The nutrient concentrations in this ancient and deeply weathered landscape are typically very low, especially for phosphorus, which limits plant growth despite several adaptations that maximize the efficiencies of phosphorus uptake and use (Lambers *et al.*, 2011; Lambers *et al.*, 2015; Viscarra Rossel and Bui, 2016). As much as practically possible, sites were selected to feature similar topographies and community compositions.

### Climate and edaphic characterization

Climate conditions were quantified for soil microclimate, as well as local atmospheric climate conditions for each site. Hydrological (soil water content at 10-cm depth) and thermal conditions were measured through three TMS moisture and temperature loggers (TMS4, TOMST Ltd, Prague, Czech Republic) positioned in random locations at each site and active for the duration of the study, over October, November and December of 2021. Local climate data (e.g. cumulative rainfall) for each site were also obtained from weather station data available through the Bureau of Meteorology. We extracted mean annual temperature (BIO1, MAT) and mean annual precipitation (BIO12, MAP) data for each site (Fig. 2) from WorldClim version 2.1 at a spatial resolution of 30 arc-seconds (Fick and Hijmans, 2017). Atmospheric vapour pressure deficit (VPD_a_, kPa) and relative humidity (RH%) were captured using a LI-6400XT portable gas exchange system (LI-COR Biosciences, Lincoln, NE, USA) and averaged across months for each site, by taking the ‘reference’ measures of the atmospheric air that was taken in through the gas exchange system with desiccant column on ‘full bypass’.

Edaphic conditions were characterized by soil physicochemical properties of five samples from each site. Soil samples were collected at 0- to 10-cm depth from areas within 1 m of where *S. hispidum* individuals were present, representing the active rooting zone of *S. hispidum* plants. Soil samples were dried and sieved to 2-mm particle size whereby larger rock fragments and woody debris >2 mm were removed from samples. After sieving, chemical properties were determined by CSBP Soil and Plant Analysis Laboratory (Bibra Lake, WA) using methods presented in Rayment and Lyons (2011). The edaphic traits measured include ammonium nitrate (NH_4_NO_3_, mg kg^−1^; Rayment and Lyons method 7C2b), Colwell phosphorus (P, mg kg^−1^; Rayment and Lyons method 9B), Colwell potassium (K, mg kg^−1^; Rayment and Lyons method 18A1), sulphur (S; mg kg^−1^; Rayment and Lyons method 10D1), soil pH in H_2_O (Rayment and Lyons method 4A1) and CaCl_2_ (Hallmark *et al.*, 1983), electrical conductivity (dS m^−1^; Rayment and Lyons method 3A1), trace elements including copper (Cu, mg kg^−1^), zinc (Zn; mg kg^−1^), manganese (Mn; mg kg^−1^), iron (Fe; mg kg^−1^) via diethylenetriaminepentaacetic acid (DTPA) extraction (Rayment and Lyons method 12A1), boron (B, mg kg^−1^; Rayment and Lyons method 12C2), OC (%; modified Rayment and Lyons method 6B1), exchangeable aluminium (Al, meq/100 g; Rayment and Lyons method 15G1), along with other exchangeable cations, including calcium (Ca; meq/100 g), magnesium (Mg, meq/100 g) and sodium (Na, meq/100 g), using a 1:5 soil/water extraction (modified Rayment and Lyons Method 5A4). Calcium/magnesium ratio, effective cation exchange capacity and exchangeable sodium percentage were also calculated.

### Plant morphological and physiological traits

Field measurements were conducted in October, November and December 2021. Each month, morphological and physiological traits were measured across at least 10 reproductively mature plants at each site that had grown to a rosette diameter of at least 2 cm and separated from other sampled plants by at least 2 m. Morphological traits measured include rosette size (product of the perpendicular diameters; length, width and height; cm^3^), number of flowers and plant height (cm). All morphological traits were paired with the physiological measurements from the same plant, and no plant was repeatedly measured.

Physiological performance was characterized by photosynthetic CO_2_ assimilation rate (*A*), stomatal conductance (*g_s_*), transpiration (*E*) and leaf temperature using a LI-6400XT portable gas exchange system (LI-COR Biosciences, Lincoln, NE, USA) that was equipped with a 6400-15 Extended Reach 1-cm Chamber receiving ambient light and temperature conditions. The size and morphology of *Stylidium* rosettes limit the use of other larger chambers. Measurements were taken with chamber conditions set to carbon dioxide (CO_2_) concentrations of 400 μmol CO_2_ mol^−1^; RH and temperature were maintained at ambient conditions to reflect site seasonal conditions for climate. Rosette leaves were displayed as fans upon insertion into the leaf chamber, ensuring minimal overlap between leaves. Measurements were taken when stability was reached, determined via visual analysis of real-time response curves accompanied by system stability indicators. At least three measurements were taken per plant. All gas exchange measurements were adjusted by the leaf area, by capturing a bird’s eye photograph of the leaves within the chamber and through pixel analysis using ImageJ. After gas exchange measurements were obtained, intrinsic WUE was calculated as *A/g_s_*.

Chlorophyll content was measured using a CCM-300 Chlorophyll Content Meter (Opti-Sciences, Hudson, NH, USA) by placing the sensor head directly on the leaf surface of each plant for three independent leaves per plant. Chlorophyll fluorescence (F_v_/F_m_) was captured using a Pocket PEA chlorophyll fluorometer (Hansatech Instruments Ltd, UK) with photosynthetically active radiation (PAR) set to 3500 μmol m^−2^ s^−1^ following at least 10 minutes of dark adaptation on leaves. All physiological measurements were constrained to a sampling period between 9:00 am and 12:00 pm, representing the time when the plants were most photosynthetically active, and conducted within the same sampling window (i.e. all sites were sampled within five consecutive days for each month of sampling).

### Statistical analysis

#### Patterns of latitudinal variation

All statistical analyses and ordinations were performed in the R statistical environment (version 4.1.3) using RStudio Version 2022.02.0 (R Development Core Team, 2022). Summary statistics are presented as means ± standard error for site-level differences.

Differences in morphological and physiological traits within (temporal) and between (spatial) sites were assessed using two-way analysis of variance (ANOVA) and post hoc Tukey tests using the *anova* and *TukeyHSD* functions in the *‘stats’* package, respectively. Prior to analysis, data were assessed for homogeneity of variance using the *leveneTest* function in the *‘car’* package, and data were log transformed where appropriate to meet the assumptions for the ANOVA. Phenotypic variation was estimated using the quartile coefficient of dispersion (QCD), calculated as $\left(\left[Q3-Q1\right]/\left[Q1+Q3\right]\right)$ for each trait per population for each sampling month. This statistic is suggested to be a robust alternative to the coefficient of variation, which has been found to underestimate intraspecific trait variability, or plasticity, in approximately 50% of cases (Rosas *et al.*, 2019; Hurtado *et al.*, 2020; Yang *et al.*, 2020).

A Bray–Curtis dissimilarity matrix was used for a non-parametric permutational multivariate ANOVA (Anderson, 2005) to identify significant differences between trait assemblages along the latitudinal gradient using the *adonis* function in the ‘*vegan*’ statistical package. *Post hoc* pairwise comparisons between trait assemblages were conducted to identify the multivariate differences between sites using the *pairwise.perm.manova* function in the ‘*RVAideMemoire*’ package (Anderson, 2014; Hervé and Hervé, 2020).

Principal components analysis (PCA) was used to identify the major dimensions of variation in the trait, climate and soil datasets and visualize multidimensional variation among populations and sites using the *PCA* function in the ‘*FactoMineR’* package (Lê *et al.*, 2008). The PCA was carried out on log-transformed data, and trait correlations with the first two principal components were assessed using the *dimdesc* function.

#### Climatic and edaphic associations

Redundancy analysis (RDA) was used to construct an ordination of the variation in trait composition as constrained by the edaphic and climatic variables and identify dimensions of trait variability that correlate with environmental variables using the *rda* function in the *‘vegan’* statistical package (Oksanen *et al.*, 2007). Beginning with the intercept-only model, we applied a stepwise selection procedure using the *ordistep* function to determine the most parsimonious subset of explanatory environmental variables for both the edaphic and climate models. Model choice was based on Akaike’s Information Criterion as a measure of model parsimony (Sakamoto *et al.*, 1986). Pearson correlation coefficients and variance inflation factors were assessed to avoid unnecessary redundancies in the final suite of explanatory variables using the *vif.cca* function. Models and individual axes were tested for significance using the *anova.cca* function. Vectors were superimposed onto RDA biplots with significance established through 999 permutations using the *envfit* function, also in the ‘*vegan’* statistical package (Oksanen *et al.*, 2007). We log_10_ transformed trait variables of stomatal conductance and rosette size to meet assumptions of homogeneity of variance, and all variables were centred and standardized prior to RDA to provide a comparable unitless scale to compare the trait dataset with climatic and edaphic variables.

To partition the effects of climatic and edaphic factors on trait compositions, we ran three different RDAs: a full model including both climatic and edaphic explanatory variables identified in the forward selection procedure, a partial model of climatic variables while controlling for edaphic effects and a partial model of edaphic variables while controlling for climatic effects. The unique and joint effects of climate and soil for explaining the variation in each morphological and physiological traits were then quantified using variation partitioning (Legendre and Legendre, 2012). The shared variance accounted for by both climatic and edaphic factors was calculated as the difference between the sum of the adjusted *R*^2^ for soil and climate and the adjusted *R*^2^ of the full model encompassing all factors. Marginal significance of individual explanatory variables was established through 999 permutations using the *anova.cca* function in the ‘*vegan*’ statistical package (Oksanen *et al.*, 2007). Partial RDAs were used to determine the proportion of variance that was individually constrained by the explanatory variables and the proportion that was ‘conditioned’ by all remaining variables from which we calculated the proportion of shared variance for each explanatory variable.

## Results

### Patterns of latitudinal variation

#### Climate factors

Sites along the latitudinal gradient differed significantly in all climatic traits both spatially and temporarily. Mean ambient temperature (spatial: *F*_4,30_ = 16.2, *P* < 0.001; temporal: *F*_2,30_ = 711.9, *P* < 0.001), temperature variability (spatial: *F*_4,30_ = 6.01, *P* = 0.001; temporal: *F*_2,30_ = 39.4, *P* < 0.001), maximum temperature (spatial: *F*_4,30_ = 4.63, *P* = 0.004; temporal: *F*_2,30_ = 84.9, *P* < 0.001) and minimum temperature (spatial: *F*_4,30_ = 35.0, *P* < 0.001; temporal: *F*_2,30_ = 224.7, *P* < 0.001) were all significantly higher in the northern sites (Supplementary Table S1). Additionally, thermal factors also showed significant increases along the temporal gradient during the survey period, showcasing the expected warming moving into austral summer.

Hygric factors including volumetric soil moisture (spatial: *F*_4,30_ = 2.99, *P* = 0.035; temporal: *F*_2,30_ = 36.4, *P* < 0.001) and RH (spatial: *F*_4,199_ = 38.4, *P* < 0.001; temporal: *F*_2,199_ = 45.9, *P* < 0.001) both varied significantly spatially and temporarily, with southern sites typically reflecting wetter environments with greater soil moisture availability (Supplementary Table S1). Soil moisture variability, however, only differed significantly between months (spatial: *F*_4,30_ = 1.17, *P* = 0.341; temporal: *F*_2,30_ = 35.1, *P* < 0.001).

Atmospheric VPD (spatial: *F*_4,199_ = 27.3, *P* < 0.001; temporal: *F*_2,199_ = 46.4, *P* < 0.001) and PAR (spatial: *F*_4,199_ = 6.75, *P* < 0.001; temporal: *F*_2,199_ = 3.49, *P* = 0.032) both showed significant spatial and temporal variation. Northern sites generally reflected higher VPDs, which increased moving into austral summer and PAR values were significantly higher in the northern sites, while presenting a slight decline in December (Supplementary Table S1).

#### Edaphic factors

Significant differences between sites were observed for all macronutrients: ammonium nitrate (*F*_4,20_ = 8.79, *P* < 0.001), phosphorus (*F*_4,20_ = 5.25, *P* = 0.004) and potassium (*F*_4,20_ = 13.9, *P* < 0.001) along with sulphur (*F*_4,20_ = 6.01, *P* = 0.002), magnesium (*F*_4,20_ = 3.43, *P* = 0.027) and calcium (*F*_4,20_ = 3.78, *P* = 0.019). Of the trace elements, site level differences were only present for zinc (*F*_4,20_ = 3.45, *P* = 0.027) and boron (*F*_4,20_ = 7.11, *P* < 0.001). Significant differences between sites were also observed for the salinity metrics of sodium content (*F*_4,20_ = 16.5, *P* < 0.001) and electrical conductivity (*F*_4,20_ = 6.16, *P* = 0.002). *Post hoc* comparisons highlighted that these results were almost exclusively driven by a single site, John Forrest National Park, which presented significantly higher macronutrient availabilities, salinity and the trace elements (Supplementary Table S1). Soil acidity also showed spatial differences with the three most northern sites presenting significantly higher pH levels than the two most southern sites (*F*_4,20_ = 3.09, *P* = 0.039).

#### Plant trait means and variability

**Figure 3:**
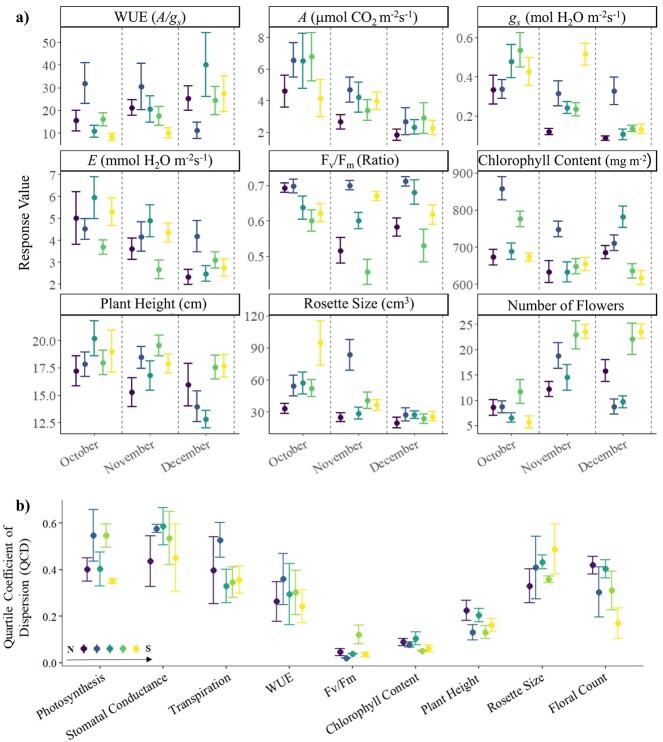
**a)** Spatiotemporal variation in functional traits across at least 10 *S. hispidum* plants (mean ± standard error) for each site along the surveyed latitudinal gradient by sampling month. Error bars, from left to right, correspond to sites from north to south—Julimar State Forest, John Forrest National Park, Bungendore Park, State Forest (Dwellingup), State Forest (Hoffman). **b)** Intraspecific trait variation as measured by the QCD across the latitudinal gradient. Error bars (mean QCD ± standard error), from left to right, correspond to sites from north (N) to south (S)—Julimar State Forest, John Forrest National Park, Bungendore Park, State Forest (Dwellingup), State Forest (Hoffman).

Overall, stomatal conductance differed between populations with the northern-most population presenting decreased stomatal conductance compared to the southern populations (*F*_4,167_ = 7.89, *P* < 0.001). Photosynthetic rate along the latitudinal gradient loosely followed a quadratic trend, though there were only marginal differences between populations (*F*_4,167_ = 2.29, *P* = 0.061). Additionally, there was a decreasing trend for both these traits across monthly sampling (photosynthesis: *F*_2,171_ = 17.1, *P* < 0.001; conductance: *F*_2,167_ = 33.1, *P* < 0.001; Fig. 3a). There were no fixed effects of population or sampling month on WUE (population: *F*_4,152_ = 1.36, *P* = 0.247; month: *F*_2,152_ = 0.57, *P* = 0.566). Transpiration was marginally different between sites (*F*_4,154_ = 2.42, *P* = 0.051; Fig. 3a), but trait means displayed significant decrease across monthly sampling (*F*_2,154_ = 14.6, *P* < 0.001; Fig. 3a). Chlorophyll content and quantum yield (F_v_/F_m_) both differed significantly across populations (content: *F*_4,196_ = 15.1, *P* < 0.001; F_v_/F_m_: *F*_4,196_ = 8.08, *P* < 0.001; Fig. 3a), with chlorophyll content peaking in the second most northern site, followed by a decrease into the southern populations. Though F_v_/F_m_ responses differed between sites, there were no distinct latitudinal patterns. Chlorophyll content displayed significant decreases across monthly sampling (*F*_2,196_ = 12.3, *P* < 0.001), but F_v_/F_m_ was statistically indistinguishable between sampling months (*F*_2,196_ = 1.31, *P* = 0.273; Fig. 3a).

**Figure 4:**
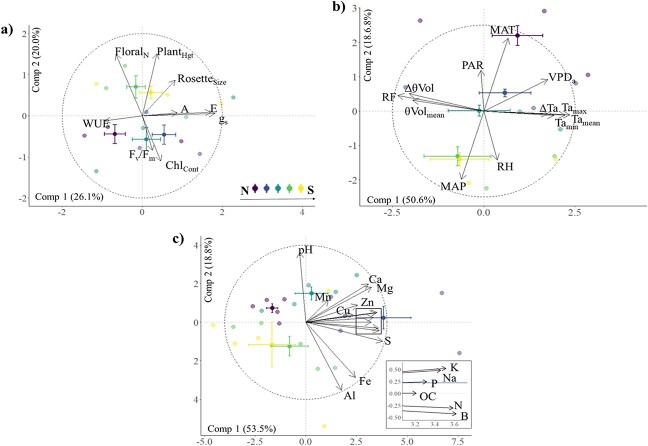
PCA biplots showing the multidimensional differences in **a)** functional traits of *S. hispidum,***b)** climate proxies and **c)** soil chemical profiles across a local latitudinal gradient. Data are presented as centroids and standard errors. The proportion of variance explained by each component axis appears in parentheses within each biplot, while axes labels correspond to the dominant gradient of variation. In **c),** the inset provides ease of visualization for clustered vectors. Survey sites, from north (N) to south (S)—Julimar State Forest, John Forrest National Park, Bungendore Park, State Forest (Dwellingup), State Forest (Hoffman). Refer to methods for units for each trait. Vector loadings for functional traits correspond to photosynthesis (*A*), transpiration (*E*), stomatal conductance (*g_s_*), WUE (defined as *A/g_s_*), maximum quantum yield of Photosystem II (F_v_/F_m_), chlorophyll content (Chl_cont_), number of flowers (Floral_N_), plant height (cm; Plant_Hgt_) and rosette size (cm^3^; Rosette_Size_). Vector loadings for climate factors correspond to MAT (°C), MAP (mm), mean RH (%), mean atmospheric VPD (kPa), mean volumetric soil moisture (%; θVol_mean_), volumetric soil moisture variability (∆θVol_mean_), mean ambient temperature (°C, Ta_mean_), minimum temperature (°C, Ta_min_), maximum temperature (°C, Ta_max_), temperature variability (°C, ∆Ta) and mean PAR and cumulative rainfall during the survey period (mm; RF). Vector loadings for edaphic factors is based on soil pH (pH), ammonium nitrate (N; NH_4_NO_3_), phosphorus (P), sodium (Na), potassium (K), sulphur (S), magnesium (Mg) and calcium (Ca), copper (Cu), zinc (Zn), manganese (Mn), iron (Fe), aluminium (Al), boron (B) and organic carbon (OC).

Morphological traits, including rosette size (*F*_4,196_ = 6.72, *P* < 0.001), plant height (*F*_4,196_ = 3.08, *P* = 0.017) and floral production (*F*_4,196_ = 10.1, *P* < 0.001), all increased with latitude, with plants being, on average, larger and taller with greater floral displays in southern populations (Fig. 3a). However, temporal trends generally showed a decrease in rosette size moving into the warmer months (*F*_2,196_ = 20.0, *P* < 0.001; Fig. 3a), consistent with rosette contraction and curling. Floral production reflected population flowering phenology, with the greatest number of flowers present in warmer months (*F*_2,196_ = 34.3, *P* < 0.001; Fig. 3), following the full development of all panicles.

Significant interaction effects of population and sampling month were only observed for WUE (*F*_8,152_ = 2.04, *P* = 0.044), stomatal conductance (*F*_8,167_ = 4.67, *P* < 0.001), chlorophyll content (*F*_8,196_ = 5.92, *P* < 0.001), rosette size (*F*_8,196_ = 2.02, *P* = 0.044) and floral production (*F*_8,196_ = 5.39, *P* < 0.001). Despite the significant population differentiation in most of the functional trait means, trait variability (QCD) was remarkably similar across the latitudinal gradient (*F*_4,130_ = 0.48, *P* = 0.751; Fig. 3b).

#### Multivariate analysis

Leaf traits varied significantly both spatially and temporally (spatial: *Pseudo-F*_1,136_ = 8.00, *P* < 0.001; temporal: *Pseudo-F*_1,136_ = 26.99, *P* < 0.001; Fig. 4a). Similarly, climate factors also showed significant spatiotemporal variation (spatial: *Pseudo-F*_1,136_ = 44.8, *P* < 0.001; temporal: *Pseudo-F*_2,44_ = 352.7, *P* < 0.001; Fig. 4b). Edaphic conditions varied spatially (*Pseudo-F*_1,138_ = 23.3, *P* < 0.001; Fig. 4c).

The first two components of the PCA describing variation in functional traits accounted for 46.1% of variation across the latitudinal gradient (Fig. 4a). The first axis, which was characterized primarily by a gas exchange gradient, accounted for 26.1% of this variation. The leaf traits that displayed the strongest positive correlations to the first dimension were stomatal conductance (*R*^2^ = 0.91, *P* < 0.001) and transpiration (*R*^2^ = 0.85, *P* < 0.001), respectively, while WUE (*A*/g_s_; *R*^2^ = 0.46, *P* < 0.001) presented the strongest negative correlation to the first axis. The second axis accounted for 20.0% of the trait variation. However, this axis was characterized by a morphological gradient with the floral counts (*R*^2^ = 0.76, *P* < 0.001), plant height (*R*^2^ = 0.75, *P* < 0.001) and rosette size (*R*^2^ = 0.42, *P* < 0.001) presenting the strongest positive correlations. Chlorophyll content (*R*^2^ = 0.55, *P* < 0.001) and F_v_/F_m_ (*R*^2^ = 0.42, *P* < 0.001) presented the strongest negative correlations with the second axis.

The PCA describing climate cumulatively described 69.2% of variation with the first two principal component axes (Fig. 4b). The first axis, representing local microclimate, accounted for 50.6% of this variation while the second accounted for 18.6%, representing long-term climate averages. The first axis was positively associated with the mean ambient temperature as constrained by the survey period (*R*^2^ = 0.96, *P* < 0.001), followed closely by local maximum (*R*^2^ = 0.92, *P* < 0.001), and negatively associated with soil moisture variability (*R*^2^ = 0.92, *P* = 0.001) and rainfall events as constrained by the survey period (*R*^2^ = 0.91, *P* = 0.001). The second axis was positively associated with MAT (*R*^2^ = 0.83, *P* < 0.001) and negatively associated with MAP (*R*^2^ = 0.81, *P* < 0.001).

The first two components of the PCA describing variation in the edaphic environment across the gradient accounted for a cumulative 72.3% of variation (Fig. 4c). The first axis component, which reflects a gradient of macro- and micronutrient availability, accounted for 53.5% of this variation. Sulphur (*R*^2^ = 0.94, *P* < 0.001) presented the strongest positive association with the first component, closely followed by boron, ammonium nitrate, potassium, sodium and phosphorus, respectively (*R*^2^ range = 0.82–0.90, *P* < 0.001). The second principal component axis accounted for 18.8% of the variation. However, this axis corresponded most strongly to a trace metal and acidity gradient, which was significantly positively associated with soil pH (*R*^2^ = 0.91, *P* < 0.001) and negatively associated with aluminium (*R*^2^ = 0.88, *P* < 0.001) and iron (*R*^2^ = 0.71, *P* < 0.001) concentrations.

### Climatic and edaphic associations

#### Climate factors

**Figure 5:**
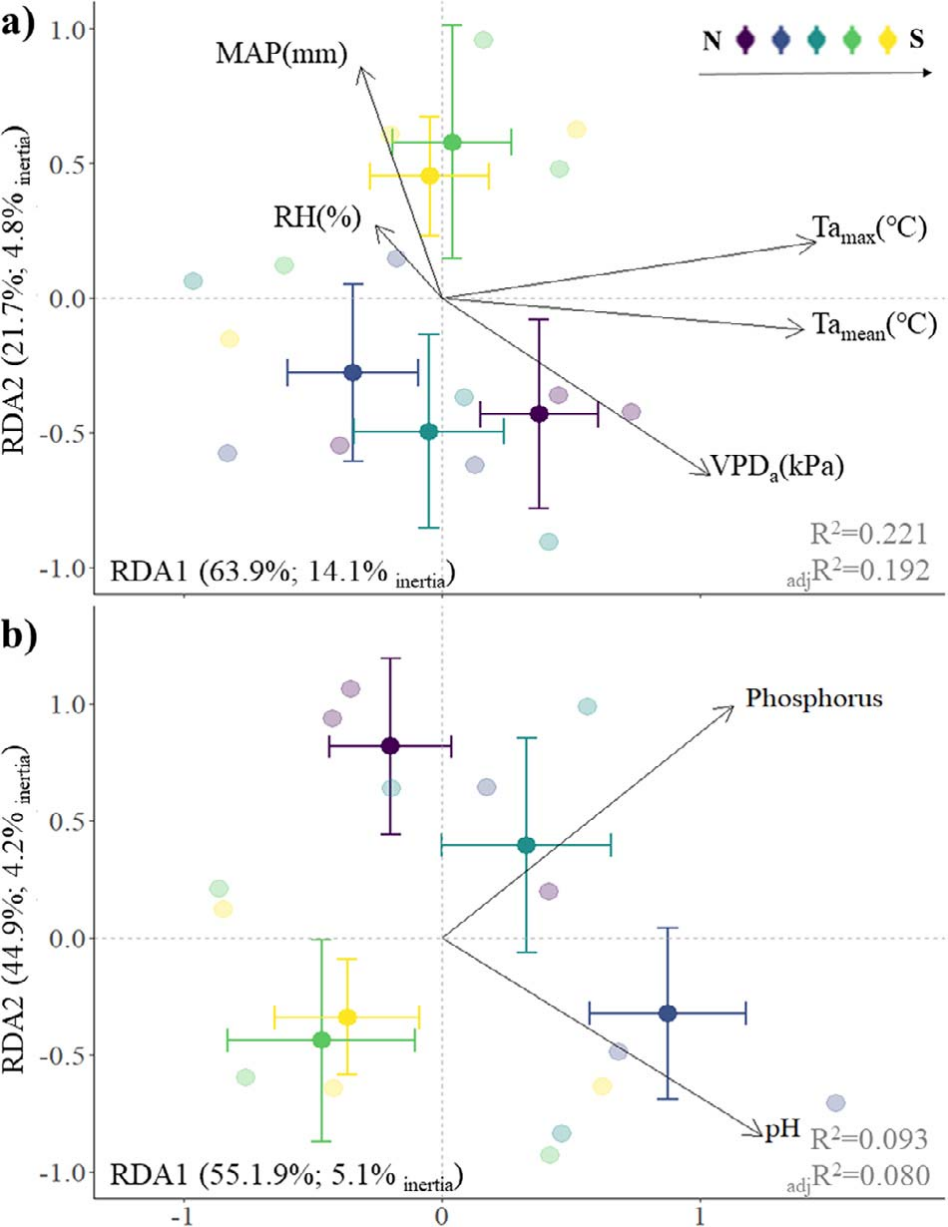
RDA biplots showing plant functional trait composition as constrained by **a)** climatic factors and **b)** edaphic factors. Opaque points (±95% confidence intervals) in panels **a)** and **b)** represent site centroids, showcasing the latitudinal variation of survey sites, from north (N) to south (S)—Julimar State Forest, John Forrest National Park, Bungendore Park, State Forest (Dwellingup), State Forest (Hoffman). The proportion of variance explained by each axis appears in parentheses, followed by the proportion of inertia explained. *R*^2^ and adjusted *R*^2^ (_adj_*R*^2^) are overlayed in grey text. The climatic and edaphic factors used in these analyses were the ones revealed to be the most relevant following a forward selection procedure using the *ordistep* function in the R statistical package *‘vegan’*. Vector labels correspond to mean atmospheric VPD (kPa), mean maximum temperatures (°C, Ta_max_), mean ambient temperature (°C, Ta_mean_), MAP (mm), RH (%), soil pH (pH) and phosphorus content.

We used RDA to understand how the multivariate structure of leaf traits can be explained by the climate characteristics at each site. Following the forward selection procedure, only five factors were identified for inclusion in the final model: mean ambient temperature (°C), mean maximum temperature (°C), RH (%), mean atmospheric VPD (kPa) and MAP (mm). The RDA model of climatic effects explained a significant portion (22.1%) of the weighted variance (inertia) in plant traits (*F*_5*,*136_ = 7.71, *P =* 0.001), and a cumulative 85.6% of variance in the weighted averages and class totals was accounted for by the first two axes (RDA1: *Pseudo-F*_1*,*136_ = 21.7, *P =* 0.001; RDA2: *Pseudo-F*_1*,*136_ = 8.35, *P =* 0.001; Fig. 5a, Table 1a). Analysis of individual terms indicated that the strongest climatic association with leaf trait data was mean maximum temperature (adjusted *R*^2^ = 0.126, *F*_1*,*140_ = 23.8, *P =* 0.001; Table 1b), followed by mean ambient temperature (adjusted *R*^2^ = 0.118, *F*_1*,*140_ = 22.4, *P =* 0.001; Table 1b), atmospheric VPD (adjusted *R*^2^ = 0.077, *F*_1*,*140_ = 15.0, *P =* 0.001; Table 1b), MAP (adjusted *R*^2^ = 0.018, *F*_1*,*140_ = 4.53, *P =* 0.002; Table 1b) and RH (adjusted *R*^2^ = 0.011, *F*_1*,*140_ = 3.16, *P =* 0.01; Table 1b).

**Table 1:** **a)** Results of RDA on trait compositions of *S. hispidum* Lindl. Models partitioned inertia (variance) into that encompassed by climate and soil factors, as well as a partial model of climatic variables while controlling for edaphic effects (Climatic | Edaphic), a partial model of edaphic variables while controlling for climatic effects (Edaphic | Climatic) and a joint model capturing the shared inertia between both climatic and edaphic models (Climatic ∩ Edaphic) with asterisks denoting model significance (^*^, *P* < 0.05; ^**^, *P* < 0.01; ^***^, *P* < 0.001, NA = Not Available). **b)** Partitioned inertia by simple RDAs of independent climatic and edaphic factors. Proportion constrained corresponds to the variance constrained by the sub-models (in a) and independent factors (in b) relative to the proportion constrained by the full model. Asterisks denote marginal (Type III) effects of individual explanatory variables in a model with all other terms accounted for.

**Model**	**Partitioned inertia**	**Proportion constrained**	**% Relative**	_**adj**_ ***R*** ^**2**^	**F(*P*)**
(a) Summary of RDA model results for plant trait composition variation					
Climatic	1.988	0.221	87.155	0.192	7.71^***^
Edaphic	0.841	0.093	36.870	0.080	7.16^***^
Climatic | edaphic	1.440	0.160	63.130	0.134	5.74^***^
Edaphic | climatic	0.293	0.033	12.845	0.022	2.92^***^
Climatic ∩ Edaphic	0.548	0.061	24.025	0.058	NA
Full	2.281	0.253	100%	0.214	6.49^***^
Total inertia	9.000				
(b) Independent factor contributions to full RDA model and marginal effects					
Ta_max_ (°C)	1.193	0.133	52.307	0.126	2.996 ^**^
Ta_mean_ (°C)	1.121	0.125	49.159	0.118	2.818 ^**^
VPD_a_ (kPa)	0.752	0.084	32.962	0.077	1.647
MAP (mm)	0.227	0.025	9.963	0.018	1.551
RH (%)	0.159	0.018	6.968	0.011	2.894 ^*^
pH	0.426	0.047	18.671	0.041	1.061
Phosphorus	0.436	0.048	19.109	0.042	1.520

#### Soil factors

In the RDA for leaf traits and soil characteristics, only two factors, soil pH and phosphorus content, remained in the model after the forward selection procedure. The RDA model constructed for the edaphic drivers explained 9.3% (*R*^2^ = 0.093, adjusted *R*^2^ = 0.080) of the weighted variance (inertia) in the leaf trait data, as constrained by phosphorus and soil pH (RDA1: *Pseudo-F*_1*,*139_ = 7.89, *P =* 0.001; RDA2: *Pseudo-F*_1*,*139_ = 6.44, *P =* 0.001; Fig. 5b, Table 1a). Analysis of individual terms indicated that both phosphorus (adjusted *R*^2^ = 0.042, *F*_1*,*140_ = 8.69, *P =* 0.001; Table 1b) and soil pH (adjusted *R*^2^ = 0.041, *F*_1*,*140_ = 8.49, *P =* 0.001; Table 1b) accounted for a similar amount of variation in the leaf trait data.

#### Variance partitioning

**Figure 6:**
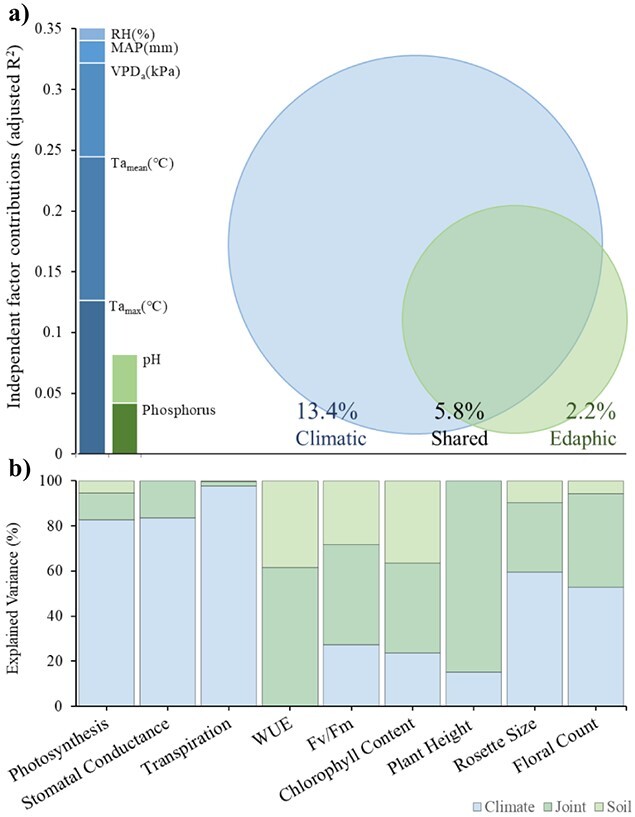
**a)** Variance partitioning of plant functional traits between the independent effect of climatic factors, the independent effect of edaphic factors and the effect of climatic and edaphic factors in combination. Independent contributions of climatic and edaphic factors are presented in the stacked bar graph and reflect adjusted *R*^2^ contributions. The climatic and edaphic factors used in these analyses were the ones revealed to be the most relevant following a forward selection procedure using the *ordistep* function in the R statistical package *‘vegan’*. **b)** Results of partitioning variation driven independently by climate and soil factors as well as their joint effects for all functional traits. Refer to methods for unit measures for each trait.

Variance partitioning showed that overall, climate factors, as determined by the forward selection procedure, explained more variation (adjusted *R*^2^ = 0.192; Table 1a) in the collective matrix of leaf traits than did soil (adjusted *R*^2^ = 0.080; Table 1a). A partial model of climatic variables while controlling for edaphic effects captured more variation (adjusted *R*^2^ = 0.134; Table 1a) in the plant trait dataset than did the partial model of edaphic variables while controlling for climatic effects (adjusted *R*^2^ = 0.022; Table 1a). Climatic and edaphic factors had a shared explained variance of 5.8% (adjusted *R*^2^ = 0.058; Fig. 6a, Table 1a). Partitioning out the effects of climate, soils and their joint effect for each independent plant trait showcased between 15.1% and 97.6% of the explained variation observed within each trait was captured by the independent effects of climate, and up to 38.5% of the variance was captured by the independent effects of soil (Fig. 6b). Joint contributions ranged between 2.02% and 84.9% (Fig. 6b). Collectively, climate captured the most variance in all traits except for WUE, whereby climate effects were captured by joint contributions with edaphic factors.

## Discussion

Examining where morphological and physiological trait diversity exists, and the environmental factors associated with ITV, can provide insight into how plants are performing in current conditions and their resilience to environmental change. Our research details the physiological and morphological variation within and between five spatially distinct *S. hispidum* populations and the associations with local climatic and edaphic conditions. There were marked differences in the thermal and hygric conditions along a latitudinal gradient, confirming that northern sites were hotter and drier than southern sites. We found that northern populations presented significantly reduced gas exchange, rosette size and floral investment compared to the southern populations, despite the similar patterns of trait variability (QCD) across populations. Trait expression in the northern populations is likely a result of morphological and physiological strategies that work to cope with heat and drought stress. Overall, climate factors, and in particular, temperature, accounted for the greatest proportion of variation in independent morphophysiological traits, as well as multivariate trait composition.

### Patterns of trait variation

Conforming to our hypothesis, that trait means would vary along the gradient, we found northern populations to present trait means reflective of water conservation strategies such as reduced stomatal conductance, smaller rosettes and limited floral investment compared to the southern populations. Generally, these patterns of form and function across the latitudinal gradient were captured by two main axes of variation (Fig. 4a), with the first axis broadly reflecting a gas-exchange gradient and the second reflecting a productivity and morphological gradient. Such axes have emerged in various forms across several previous studies. For example, both Díaz *et al.* (2016) and Joswig *et al.* (2022) conclude that two main axes of variation include size and leaf economics across global plant trait datasets. Along a local elevational gradient, Visakorpi *et al.* (2022) define a physiological gradient, based on WUE and gas exchange, and a morphological gradient, based on measures such as plant width and leaf area. Collectively, these findings support the notion that axes of variation relating to physiology, economics and morphology persist at local intraspecific and global interspecific scales (Joswig *et al.*, 2022). Therefore, morphophysiological trait data may be used to generate predictions of how species may respond to changing environments to gain insights into the mechanisms driving trait–environment relationships.

Intraspecific variation is a key component of functional trait diversity, and adaptive plasticity is a major mechanism by which plants respond to abiotic and biotic stress (Gratani, 2014). High ITV is considered to promote species acclimation, increase competitive capacity and population persistence in dynamic environments and may be a key index for predicting population and species resilience in the face of a changing climate or increasingly degraded landscapes (Valladares *et al.*, 2007; Valladares *et al.*, 2014). However, we found that trait variability was evenly distributed along the latitudinal gradient with all populations presenting statistically indistinguishable differences in the QCD. Consequently, our results do not support the prediction that northern populations would reflect reduced trait variability, although this finding is consistent with previous studies that report no evidence to suggest that there is population differentiation in intraspecific trait variability (Matesanz *et al.*, 2012; Kühn *et al.*, 2021). While we do not distinguish between the trait variation that arises from heritable genetic differences or by phenotypic plasticity, the similarities in trait variability among populations may suggest that there is no differential selection for plasticity across the latitudinal gradient, or there is insufficient heritable variation to drive population differentiation in trait variability for *S. hispidum* (Goldstein and Ehrenreich, 2021). It could also be suggested that the similar levels of population level variability are the result of similar degrees of heterogeneity, despite different means, in abiotic conditions at different sites (De Kort *et al.*, 2020). However, these conclusions are context dependent and depend largely on the species, environmental heterogeneity and the extent of a species distribution.

### Drivers of latitudinal variation

Generally, climatic and edaphic gradients are associated with significant differences in functional traits, relating to the morphology, physiology and reproduction of plants, and they are recognized as important predictors of population persistence in the face of environmental change (Violle *et al.*, 2007). We found marked differences in the thermal and hygric conditions along the latitudinal gradient, such that northern sites were 1.6°C warmer than southern sites, on average, with maximum temperatures reaching 3.3°C higher, while there was a 99% increase in mean rainfall from the driest northern site to the wettest southern site during the survey period alone. Moreover, we observed significantly higher macronutrient availability in the second most northern site, John Forrest. While the effects of climate on edaphic factors were not examined here, the patterns observed in soil factors loosely correspond to Albrecht’s conceptual model (Huston, 2012), such that soil pH, exchangeable cations and nutrient availability track a unimodal pattern from being low at the extremes for dry and wet conditions (Huston, 2012; Maire *et al.*, 2015).

RDA, together with variance partitioning, allowed the determination that climate factors consistently accounted for a significantly greater portion of the weighted variance in plant traits than that of edaphic factors. Of the climate factors defined within this study, only ambient temperature, maximum temperature, atmospheric VPD, moisture availability and rainfall presented significant associations with variance in plant functional traits along the latitudinal gradient, while only soil pH and phosphorus emerged as significant predictors out of the available soil factors. Separately, the climate factors alone constrained 22.1% (0.192 adjusted *R*^2^) of the total inertia in plant traits, while the soil factors constrained 9.3% (0.080 adjusted *R*^2^). This result contrasts with findings recorded at global scales, whereby soil predictors were found to explain more variance in individual leaf traits than climate (Ordoñez *et al.*, 2009; Maire *et al.*, 2015). However, this finding highlights the dominant role that climate can play in structuring trait expression in *S. hispidum* and suggests that future climate change scenarios may lead to trait shifts across populations in response to the effects of warming and drying. Moreover, there is uncertainty surrounding the extent of abiotic stress that this species can tolerate, highlighting the importance of characterizing physiological thresholds and resilience at biologically meaningful scales.

Despite edaphic factors being less correlated with plant traits than climate in our model species, we found that soil pH and phosphorus content explained 4.1% and 4.2% of total inertia, respectively. These results conform, in part, to those observed in a global, interspecific analysis conducted by Maire *et al.* (2015), finding the same two soil variables to have the strongest explanatory power over the variation in plant traits. While we found the explanatory power of soil pH and phosphorus availability to be substantially less than climatic variables, both soil pH and phosphorus content play key roles in shaping plant growth, resource acquisition and performance (Malhotra *et al.*, 2018; Neina, 2019). When considered across a broader gradient of soil ecotypes, increased alkalinity will often be associated with a higher availability of nutrients and reduce the acquisition costs of key macronutrients, such as nitrogen (Maire *et al.*, 2015). Increased acidity, however, can compromise root form and function and, as a consequence, inhibit CO_2_ assimilation (Long *et al.*, 2017), reduce water uptake (Bian *et al.*, 2013) and induce oxidative stress (Martins *et al.*, 2013). We observed a unimodal distribution of phosphorus content along the latitudinal gradient with intermediate populations presenting significantly greater phosphorus availability than that of marginal populations. Photosynthesis is generally reduced in phosphorus deficient soils, although there is some degree of adaptation to be expected for plants growing in environments with reduced phosphorus availability (Lambers *et al.*, 2015). Nevertheless, the unimodal patterns in photosynthesis do correspond to those of phosphorus availability, likely contributing to spatial variance observed in photosynthesis in this study.

The tendency for plants to exhibit morphological and physiological shifts along climate gradients is well known and in accordance with theory to predict shifts in gas exchange and water relations as strategies to minimize water loss and maximize water uptake whilst maintaining physiological functioning (Reich *et al.*, 2003; Westoby and Wright, 2006). We found that the strongest climate driver of variation in the leaf trait data was temperature, with mean ambient and maximum temperatures independently explaining 11.8% and 12.6% of inertia, respectively. This conforms to global scale trends in plants traits as they relate to temperature and precipitation, with Moles *et al.* (2014) finding that temperature is significantly correlated with 15 out of 21 plant traits while precipitation correlated to a lesser extent, with only six of these traits. Above-optimal temperatures and drying environments can impair plant growth, development, form and function (Bykova *et al.*, 2012), often exerting greater influences in combination than either abiotic stress does independently (Zandalinas *et al.*, 2018). Collectively, the patterns observed in plant traits observed in the northern populations can be seen as strategies to manage the economics of gas exchange and be under stronger selection pressures from climatic factors to mitigate or cope with warming and drying conditions. However, we are limited in our capacity to conclude whether the patterns of trait means are truly reflective of genetic differentiation supporting local adaptation. Therefore, future studies should seek to disentangle the relative contribution of environmental, maternal and genetic effects on plant responses (e.g. growth rates and physiological function) to combined heat and drought stress under controlled “common-garden” scenarios.

### Implications for conservation

While our results show short-term responses to interacting environmental drivers, there may be long-term implications for local extinction if trait modifications that impact plant fitness fail to keep up with rapidly changing environments. In the southwest region of Western Australia, daily maximum temperatures are expected to increase up to 2.4°C by mid-century and 4.2°C by the end of the century (Jakob *et al.*, 2012; CSIRO and BOM, 2015). Similarly, MAP is expected to decline by 17% by mid-century and 46% by the end of the century under an intermediate-emission scenario (Jakob *et al.*, 2012; Sudmeyer *et al.*, 2016). In this regard, it is possible that by the end of the century, the southern populations may present a phenotype more reflective of those currently observed in northern populations, as the latitudinal gradient represents a thermal gradient difference of 1.4°C in the warmest quarter, and 39.4% decrease in MAP. The consequences for ongoing warming and drying in the northern populations are less clear, though they may be detrimental if the adaptive capacity of the species is exceeded by the rate of change, or if tolerance thresholds for abiotic stress are surpassed. In this instance, hotter and drier conditions may result in range contractions, seeing the local extinction of northern-most populations, or potentially range shifts or expansions further south to habitats that foster milder conditions (Fitzpatrick *et al.*, 2008), especially considering that climate-related local extinctions are already widespread across the globe (Wiens, 2016). However, to date, there are no studies to report on the intergenerational effects of abiotic stress on *Stylidium,* nor any specific parameterisation of drought or thermal tolerance. Therefore, it is difficult to predict with greater certainty as to how this species will perform in future climates, highlighting a major gap in our scientific knowledge for not only the focal species of this study, but also the exceptionally diverse flora of southwest Australia.

Unless appropriate conservation and management initiatives are implemented to address the potential threat of climate change, the future remains unclear for northern marginal populations of *S. hispidum* in warming and drying southwest Western Australia. From a conservation perspective, *ex situ* storage of seed material may temporarily safeguard population genotypes and serve as a repository source for *in situ* conservation, restoration or translocation efforts (Merritt and Dixon, 2011). Additionally, it is important to monitor changes in population abundance and trait compositions as climate-associated changes unfold. If vulnerabilities are exacerbated in the future, targeted gene flow (Hufford *et al.*, 2012; Hufford *et al.*, 2016), managed relocation (Butt *et al.*, 2021; Onley *et al.*, 2021) and microclimatic amelioration (Soliveres *et al.*, 2011; Brigham and Suding, 2023) may be considered to facilitate adaptation or open migration pathways, especially for priority species. Nevertheless, it is important to note that the findings, interpretations and speculations posed in this study do not necessarily translate to other species, as the results are largely species specific and context dependent, with other trends potentially emerging at different spatial and temporal scales than those observed here. As such, ongoing conservation of at-risk species calls for investment into evidence-based insight into the intraspecific variation in tolerance to interactive stressors associated with environmental change.

## Conclusions

Here, we have demonstrated that while intraspecific trait variability may be consistent among populations spanning a latitudinal gradient*,* patterns of trait means are strongly associated with climate, and to a lesser extent, edaphic factors. While the trait–environment correlations identified in our study should not be assumed to be direct causal relationships, the dominant impact of climatic factors shaping functional trait expression along the gradient suggests that trait shifts will occur with future climate change. Disentangling the abiotic drivers of functional trait variation will be key to understanding the mechanisms underpinning local adaptation and population-level responses to ongoing environmental change. Understanding these dynamics is of global relevance; however, it is particularly important within the context of our study system, where increased temperatures and declining rainfall risk the ‘transition or collapse’ of the vulnerable Jarrah Forest ecosystems (Lawrence *et al.*, 2022).

## Supplementary Material

Web_Material_coae018

## Data Availability

The datasets generated during and/or analysed during the current study are available from the corresponding author on reasonable request.
