# Supplementary material for: Local adaptation to climate inferred from intraspecific variation in plant functional traits along a latitudinal gradient
Source: Conserv Physiol. 2024 May 5;12(1):coae018. doi: 10.1093/conphys/coae018 (PMC11074481; doi:10.1093/conphys/coae018)
Supplement: Web_Material_coae018 [file web_material_coae018.zip › Tudor_et_al_2024_-_Conservation_Physiology_SI_-_Supplementary_Information.pdf]

# Supplementary Information: Local adaptation in an endemic plant inferred from climate-driven variation in functional traits

EMILY P. TUDOR[1,2], WOLFGANG LEWANDROWKSI[2,1], SIEGFRIED KRAUSS[2,1], ERIK J. VENEKLAAS[1]

*[1]School of Biological Sciences, University of Western Australia, Nedlands, WA 6009, Australia. [2]Kings Park Science, Department of Biodiversity Conservation and Attractions, West Perth, WA 6005, Australia.*

**Table S1: Summary statistics (mean ± standard error) for climate and soil factors across five sampling locations in the Southwest of Western Australia.**

| Factor | Abv. | Julimar | John Forrest | Bungendore | Del Park | Hoffman |
|---|---|---|---|---|---|---|
| Ammonium Nitrate ($NH_4NO_3$; mg kg $^{-1}$) | **N** | 6.80 ± 0.66 | 26.2 ± 4.75 | 11.8 ± 1.24 | 11.0 ± 2.28 | 8.40 ± 5.66 |
| Colwell Phosphorus (mg kg $^{-1}$) | **P** | 4.00 ± 0.31 | 8.80 ± 0.86 | 6.20 ± 0.80 | 5.60 ± 0.51 | 5.60 ± 1.07 |
| Colwell Potassium (mg kg $^{-1}$) | **K** | 0.08 ± 0.01 | 0.35 ± 0.06 | 0.11 ± 0.02 | 0.10 ± 0.02 | 0.08 ± 0.02 |
| Sulphate Sulfur (mg kg $^{-1}$) | **S** | 5.48 ± 0.44 | 18.0 ± 3.06 | 8.48 ± 1.21 | 8.64 ± 1.67 | 7.54 ± 2.36 |
| Organic Carbon (%) | **OC** | 2.29 ± 0.19 | 3.95 ± 0.33 | 3.85 ± 0.38 | 3.22 ± 0.54 | 2.21 ± 0.66 |
| Soil pH (in $H_20$) | **pH** | 5.74 ± 0.06 | 5.76 ± 0.12 | 5.82 ± 0.02 | 5.40 ± 0.11 | 5.46 ± 0.15 |
| Copper (mg kg $^{-1}$) | **Cu** | 0.30 ± 0.02 | 0.29 ± 0.04 | 0.31 ± 0.02 | 0.27 ± 0.02 | 0.24 ±0.03 |
| Iron (mg kg $^{-1}$) | **Fe** | 27.14 ± 3.68 | 47.5 ± 5.92 | 30.8 ± 2.82 | 47.1 ± 10.4 | 40.1 ±13.6 |
| Manganese (mg kg $^{-1}$) | **Mn** | 11.25 ± 0.86 | 5.99 ± 1.85 | 15.9 ± 3.25 | 9.62 ± 2.07 | 10.9 ± 5.06 |
| Zinc (mg kg $^{-1}$) | **Zn** | 0.21 ± 0.03 | 0.64 ± 0.19 | 0.63 ± 0.22 | 0.22 ± 0.04 | 0.14 ± 0.01 |
| Boron (mg kg $^{-1}$) | **B** | 0.30 ± 0.02 | 0.74 ± 0.12 | 0.33 ± 0.06 | 0.28 ± 0.04 | 0.30 ± 0.08 |
| Aluminium (meq/100g) | **Al** | 0.32 ± 0.05 | 0.80 ± 0.22 | 0.26 ± 0.04 | 0.82 ± 0.23 | 0.79 ± 0.38 |
| Calcium (meq/100g) | **Ca** | 2.93 ± 0.33 | 5.01 ± 0.85 | 5.20 ± 1.46 | 2.14 ± 0.53 | 1.33 ± 0.82 |

| | | | | | | |
|---|---|---|---|---|---|---|
| Magnesium (meq/100g) | **Mg** | 0.94 ± 0.11 | 1.51 ± 0.27 | 1.18 ± 0.34 | 0.66 ± 0.17 | 0.39 ± 0.23 |
| Sodium (meq/100g) | **Na** | 0.08 ± 0.01 | 0.45 ± 0.08 | 0.12 ± 0.02 | 0.11 ± 0.03 | 0.08 ± 0.03 |
| Mean annual temperature (°C) | **MAT** | 17.5 ± 0.00 | 17.3 ± 0.00 | 17.1 ± 0.00 | 16.9 ± 0.00 | 16.6 ± 0.00 |
| Mean annual precipitation (mm) | **MAP** | 716 ± 0.00 | 927 ± 0.00 | 1103 ± 0.00 | 1183 ± 0.00 | 1029 ± 0.00 |
| Mean ambient temperature (monthly; °C) | $\mathbf{Ta_{mean}}$ | 19.3 ± 0.94 | 19.5 ± 1.29 | 18.5 ± 0.99 | 18.14 ± 0.94 | 17.9 ± 1.49 |
| Temperature variability (monthly; °C) | **ΔTa** | 37.6 ± 1.00 | 32.3 ± 1.35 | 34.7 ± 1.60 | 34.6 ± 1.24 | 34.8 ± 1.35 |
| Minimum temperature (monthly; °C) | $\mathbf{Ta_{min}}$ | 7.00 ± 0.45 | 8.66 ± 0.79 | 6.30 ± 0.54 | 6.64 ± 0.36 | 6.98 ± 0.59 |
| Maximum temperature (monthly; °C) | $\mathbf{Ta_{max}}$ | 44.6 ± 1.25 | 41.0 ± 1.52 | 41.0 ± 1.95 | 41.3 ± 1.48 | 41.74 ± 1.78 |
| Cumulative rainfall (monthly; mm) | **RF** | 1014 ± 332 | 670 ± 224 | 1209 ± 470 | 1332 ± 566 | 1172 ± 429 |
| Relative humidity (monthly; %) | **RH** | 43.3 ± 1.38 | 48.7 ± 1.10 | 49.6 ± 1.44 | 54.1 ± 1.53 | 48.4 ± 5.37 |
| Mean volumetric soil moisture content (θ) | $\mathbf{\theta Vol_{mean}}$ | 0.23 ± 0.02 | 0.19 ± 0.02 | 0.20 ± 0.02 | 0.23 ± 0.02 | 0.25 ± 0.02 |
| Volumetric soil moisture variability (Δθ) | **ΔθVol** | 0.17 ± 0.05 | 0.11 ± 0.03 | 0.15 ± 0.04 | 0.20 ± 0.07 | 0.15 ± 0.04 |
| Mean photosynthetically active radiation (μmol $m^{-2}s^{-1}$) | **PAR** | 773 ± 109 | 807 ± 32.5 | 945 ± 69.9 | 528 ± 137 | 468 ± 39.5 |
| Atmospheric vapour pressure deficit (kPa) | $\mathbf{VPD_a}$ | 2.74 ± 0.12 | 1.97 ± 0.13 | 2.63 ± 0.19 | 1.91 ± 0.35 | 1.71 ± 0.13 |

**Table S2: Summary statistics (mean ± standard error) for climate factors across sampling months.**

| Factor | Abv. | October | November | December |
|---|---|---|---|---|
| Mean ambient temperature (°C) | $\mathbf{Ta_{mean}}$ | 15.01 ± 0.06 | 18.80 ± 0.07 | 22.10 ± 0.10 |
| Temperature variability (°C) | **ΔTa** | 30.50 ± 0.21 | 37.52 ± 0.16 | 36.48 ± 0.28 |
| Minimum temperature (°C) | $\mathbf{Ta_{min}}$ | 5.51 ± 0.09 | 6.91 ± 0.06 | 8.87 ± 0.15 |
| Maximum temperature (°C) | $\mathbf{Ta_{max}}$ | 36.00 ± 0.23 | 44.43 ± 0.15 | 45.35 ± 0.16 |
| Mean cumulative rainfall (mm) | **RF** | 217.12 ± 8.65 | 28.31 ± 1.16 | 6.79 ± 0.96 |
| Mean volumetric soil moisture content (θ) | $\mathbf{\theta Vol_{mean}}$ | 0.28 ± 0.002 | 0.21 ± 0.003 | 0.16 ± 0.001 |
| Relative humidity (%) | **RH** | 46.8 ± 0.97 | 50.4 ± 0.79 | 43.2 ± 0.68 |
| Volumetric soil moisture variability (Δθ) | **ΔθVol** | 0.28 ± 0.01 | 0.13 ± 0.002 | 0.03 ± 0.001 |
| Mean photosynthetically active radiation (μmol $m^{-2}s^{-1}$) | **PAR** | 725 ± 34.2 | 769 ± 39.2 | 580 ± 24.7 |
| Atmospheric vapour pressure deficit (kPa) | $\mathbf{VPD_a}$ | 1.63 ± 0.06 | 2.05 ± 0.09 | 2.66 ± 0.04 |
